# Limb Preservation Using Edetate Disodium-based Chelation in Patients with Diabetes and Critical Limb Ischemia: An Open-label Pilot Study

**DOI:** 10.7759/cureus.6477

**Published:** 2019-12-27

**Authors:** Ivan Arenas, Francisco Ujueta, Denisse Diaz, Timothy Yates, Brandon Olivieri, Robert Beasley, Gervasio Lamas

**Affiliations:** 1 Cardiology, Mount Sinai Medical Center, Miami Beach, USA; 2 Internal Medicine, Mount Sinai Medical Center, Miami Beach, USA; 3 Interventional Radiology, South Beach Vascular, PLLC / Palm Vascular Centers, Delray Beach, USA; 4 Interventional Radiology, Mount Sinai Medical Center, Miami Beach, USA

**Keywords:** critical limb ischemia, peripheral arterial disease, limb amputation, chelation therapy, edetate disodium

## Abstract

Background

In 2015, there were 30.3 million patients with diabetes in the US, including 25.2% of people ages 65 or older and 108,000 hospitalizations for non-traumatic amputations. Severe diabetic limb disease includes critical limb ischemia (CLI ) due to an infrapopliteal disease with foot pain and ischemic ulcerations including gangrene. Environmentally acquired toxic metals, such as lead and cadmium, have been associated with cardiovascular disease. Thus, we designed the present unblinded pilot study to determine whether there was a signal of benefit for edetate disodium-based infusions in patients with critical limb ischemia.

Methods

This was an open-label pilot study in 10 patients with diabetes and critical limb ischemia. Each patient received up to 50 edetate disodium-based infusions and was assessed for safety, clinical efficacy, metal excretion, and quality of life. The primary endpoint was to assess the effect of edetate disodium-based therapy plus vitamins in patients with diabetes and infra-popliteal peripheral artery disease presenting with severe CLI and determine if there were improvements in vascular flow parameters.

Results

We enrolled 10 (60% male) predominantly Caucasian (90%) subjects. The mean age was 75.3 (8.0) years. Smoking was reported by 30%. There were 70% with coronary artery disease (30% had prior coronary artery bypass grafting) and 50% had a prior lower-extremity amputation, three having previous minor amputations and two major amputations. There were no major adverse cardiovascular events during the infusion phase through the one-year follow-up. Patients completing 40 infusions demonstrated complete wound healing and improvement in the quality of life.

Conclusion

Patients with diabetes and CLI treated with a regimen of edetate disodium-based infusions demonstrated a potential signal of benefit and preliminary evidence of safety. The Trial to Assess Chelation Therapy in Critical Limb Ischemia (TACT3a), a randomized double-blind, placebo-controlled clinical trial now in progress, will further test these findings.

## Introduction

Diabetes triples the risk for atherosclerosis, including coronary disease, carotid disease, and peripheral artery disease. A hallmark of diabetes is that arterial disease is diffuse, affecting not only large and medium-sized arteries, such as the epicardial coronary arteries, but also smaller vessels, such as those of the foot, with limited options for limb salvage in advanced stages. Severe diabetic limb disease includes critical limb ischemia (CLI) due to infrapopliteal disease with foot pain and ischemic ulcerations, including gangrene. CLI may progress to amputation, the patients’ most feared complication of diabetes. In 2015, there were 30.3 million patients with diabetes in the US, including 25.2% of people ages 65 or older and 108,000 hospitalizations for non-traumatic amputations [1-2]. Therefore, there is an abundant residual risk as a therapeutic target.

Environmentally acquired toxic metals, such as lead and cadmium, have been associated with cardiovascular disease [3-5]. Urine cadmium, in particular, has been associated with peripheral artery disease (PAD) and increases in parallel with the severity of PAD [4,6]. Case series in the past have suggested that edetate disodium infusion may improve PAD while small clinical trials have been less positive [7-9]. A randomized clinical trial of a potent lead and cadmium chelator in 1708 post-myocardial infarction (MI) patients reported a reduction in clinical events, most marked in patients with diabetes [10-11]. Thus, we designed the present unblinded pilot study to determine whether there was a signal of benefit for edetate disodium infusions in patients with CLI, expecting results that would either encourage further study or move us to abandon this treatment strategy for PAD.

## Materials and methods

Methods

This was an open-label pilot study in 10 patients with CLI. Each patient received up to 50 edetate disodium-based infusions and was assessed for safety, clinical efficacy, metal excretion, and quality of life. A case report was previously reported on patient 004 [12].

Study population

Patients were ≥ 50 years of age, with diabetes, and with a diagnosis of moderate or severe infra-popliteal chronic critical limb ischemia (Rutherford Clinical Severity Score 4 or 5). Exclusion criteria were women of childbearing potential, arterial insufficiency, or ulcer in the lower extremity as a result of non-atherosclerotic disease, active osteomyelitis, or deep ulceration exposing bone or tendon in the extremity, serum creatinine > 2.0 mg/dL, platelet count < 100,000/mm^3^, allergy to any study drug, symptomatic evidence of heart failure, active cigarette smoking within the last three months, abnormal liver function tests, medical condition likely to affect patient survival within four years, diseases of copper, iron, or calcium metabolism or > 5 infusions of intravenous chelation within the preceding year. The study enrolled 10 patients at our institution. The institutional review board reviewed and approved the study, and the patients provided written informed consent. The study was performed under FDA IND (67743).

Study treatment

Patients received the previously described edetate disodium-based treatment [13]. Infusions were administered through a peripheral intravenous line over three hours. Infusions contained 3 g of edetate disodium adjusted downward based on creatinine clearance, 2 g of magnesium chloride, 100 mg of procaine HCL, 2500 U of unfractionated heparin, 7 g of ascorbate, 2 mEq of KCL, 840 mg of sodium bicarbonate, 250 mg of pantothenic acid, 100 mg of thiamine, 100 mg of pyridoxine, in a volume of 500 mL. The solution was administered twice weekly for the first 20 infusions and once weekly for infusions 21 to 40. Formal study endpoints were collected at infusion 40. If the study staff thought there was an improvement, monthly infusions for an additional 10 months were offered, totaling 50 infusions in responders. All patients also received a multivitamin preparation.

Follow-up

Patients were seen at baseline and after each infusion visit. Follow-up was at one year or until the completion of 50 infusions. Patient follow-up continued without censoring if a nonfatal endpoint occurred. Noninvasive blood flow assessments were performed at baseline and completion of 20 and 40 infusions. 

Endpoints

Primary Endpoints

The primary efficacy endpoint was a change in vascular flow parameters. The primary safety endpoints were renal function from baseline to infusion 40, symptomatic hypocalcemia or hypoglycemia within eight hours of each infusion, or Class 4 heart failure within 24 hours of an infusion. 

Secondary Endpoints

Secondary efficacy endpoints consisted of a physical change in wound severity, disease-specific quality of life (the PAD Questionnaire), generic, health-related quality of life (SF-36), and urine metal levels pre and post-infusion. 

A major secondary endpoint consisted of amputations during the infusion phase. Major amputations were defined as any procedure that resulted at a level above the ankle [14]. Health Insurance Portability and Accountability Act of 1996 (HIPAA)-compliant pictures of patients’ feet were taken at baseline and following infusions 20 and 40. All images were done in a dedicated room, with the same camera, lighting, and by the same operator. Pictures are presented as is with no alterations or edits.

Skin Perfusion Pressure

The skin perfusion pressure (Sensilase® Eden Prairie, MN) for each foot was calculated from the segmental skin perfusion pressures obtained in the medial plantar, lateral plantar, and dorsal foot areas. The “target” vascular bed was defined as the plantar segment with the lowest perfusion pressure in the affected foot at the time of initial evaluation.

Peripheral Artery Disease Questionnaire and SF-36

The PAD Questionnaire is a validated, disease-specific instrument to assess the impact of PAD. Each item in the PAD Questionnaire is mapped into individual domains [15]. A lesser score indicates more physical limitation, more symptoms, lower treatment satisfaction, and worse disease-specific quality of life.

The Medical Outcomes Study Short Form 36 Item Questionnaire (SF-36) is a generic, health-related, quality of life instrument that assesses physical and emotional limitations, general health perceptions, bodily pain, social function, and changes in health status [16]. A lower score indicates less favorable health or worse function. The general health provides an average of all items in the survey.

Urine Metals

Urine metals were collected at baseline pre and post-infusion 1, 20, and 40. The urine samples were analyzed for trace elements using an Inductively Coupled Plasma-Mass Spectrometer (ICP-MS; Doctor’s Data, St. Charles, IL). Urine metals are reported as micrograms of metal per gram of creatinine to control for urine concentration.

Statistical analysis 

Baseline characteristics are summarized using percentages for categorical variables and mean and standard deviation (SD) for continuous variables. Urine metals were summarized using means and 95% confidence intervals at baseline, infusion 20, and infusion 40. The quality of life scores obtained from the PAD and SF-36 Questionnaires and skin perfusion pressures are reported using median and interquartile ranges. Three subjects did not complete the 40 planned infusions. Thus, analyses of urine metal concentrations, feet skin perfusion pressures, and quality of life at infusions 20 and 40 included only seven participants. The non-parametric Friedman test was used to compare skin perfusion pressures at baseline and follow-up and to compare quality of life scores from the PAD and SF-36 Questionnaires at baseline, infusions 20 and 40 [17]. All statistical analyses were performed using SAS software, version 9.4 (SAS Institute Inc., Cary, NC).

## Results

Baseline characteristics

**Figure 1:**
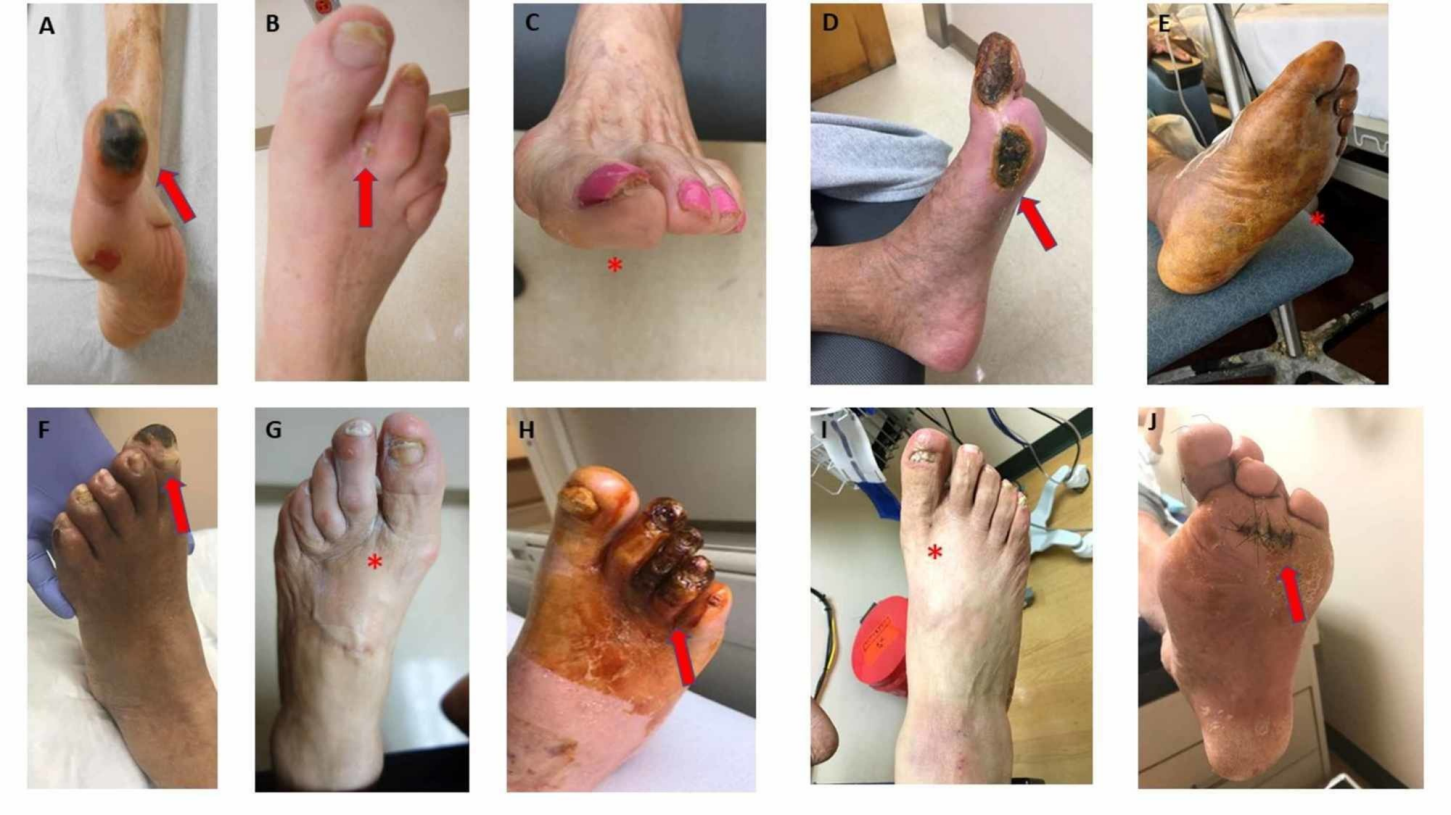
Subjects at baseline Baseline photographs of all subjects before starting edetate disodium chelation therapy. Red arrows indicate the wound at baseline. The red asterisk indicates patients with no wounds and with rest pain at baseline. (A-I) Patients 001 to 010.

We enrolled 10 (60% male) predominantly Caucasian (90%) subjects. Mean age was 75.3 (8.0) years. Previous smoking was reported by 30%. There were 70% with coronary artery disease (30% had prior coronary arterial bypass grafting), and 50% had a prior lower-extremity amputation, three having previous minor amputations, and two major amputations. All patients had a history of leg revascularization procedures. At the time of enrollment, 70% had non-healing ulcers and/or dry gangrene. All patients had ischemic limb pain at rest. Six patients had Rutherford 5 CLI severity. Six patients (60%) were categorized as stage 4 (high risk for amputation) based on the Society for Vascular Surgery wound, ischemia, and foot infection (WIfI) index (Table 1) [18]. See Figure 1 for baseline images.

**Table 1:** Baseline characteristics and urine metals Categorical values n (%); continuous values mean (std); baseline metals mean (CI); estimated Glomerular Filtration Rate (eGRF) was calculated by the Modification of Diet in Renal Disease Study equation

Variables	(n=10)
Demographics	
Age	76 ± 9
Sex, female	4 (40%)
Smoking History	3 (30%)
Comorbidities	
Hypertension	10 (100%)
Diabetes Mellitus	10 (100%)
Hyperlipidemia	6 (60%)
Cerebrovascular Events	0
Coronary Artery Disease	7 (70%)
Coronary Artery Bypass Graft	3 (30%)
Ulcer or Gangrene	7 (70%)
Peripheral Vascular History	
History of Amputations	5 (50%)
Lower Extremity Revascularizations	10 (100%)
Society of Vascular Surgery Wlfl High-risk Staging	
High Risk (clinical stage 4)	6 (60%)
Moderate Risk (clinical stage 3)	3 (30%)
Low Risk (clinical stage 2)	1 (10%)
Renal Function	
Creatinine (mg/dL)	0.92 ± 0.22
*Estimated Glomerular Filtration Rate (mL/min/1.73 m^2^)	77.6 ± 16.4
Medications	
Insulin	5 (50%)
Beta-blockers	5 (50%)
Aspirin	6 (60%)
Clopidogrel	8 (80%)
Angiotensin Converting Enzyme/Angiotensin Receptor Blocker Inhibitor	7 (70%)
Statin	7 (70%)
Baseline Urine Metals (μg of metal per g creatinine)	
Pre-infusion Cadmium	0.75 (-0.074,1.57)
Post-infusion Cadmium	4.25 (2.73,5.77)
Pre-infusion Lead	0.68 (0.24,1.12)
Post-infusion Lead	18.75(9.70,27.80)

Urine metals

All patients had urine metals measured before and after infusion 1. Urine metals were collected in seven patients at infusions 20 and 40. Urine metals increased after a single infusion of edetate disodium (Table 2). All patients had cadmium and lead detected in their baseline, pre-infusion urine. Baseline levels of urine cadmium increased by an average of 292% after the initial infusion (pre and post-infusion levels (Mean (95% CI): 0.65 μg of metal per g creatinine (0.51, 0.79) and 3.57 μg of metal per g creatinine (2.28, 4.86)), respectively, P<0.001). Baseline urine lead increased by an average of 3733% (pre and post-infusion (Mean (95%CI): 0.60 μg of metal per g creatinine (0.31, 0.89) and 23 μg of metal per g creatinine (11.53, 34.47), respectively, P<0.001) after the first edetate disodium infusion. Excretion of toxic metals was maintained throughout the 40 infusions of edetate disodium (Table 2). There was a reduction of pre-chelation urine lead of 73% and post-chelation of 68%, at baseline compared to infusion 40 (P<0.01). There were no statistically significant changes in urinary cadmium during follow-up.

**Table 2:** Urine toxic metals Urine metals at baseline and infusion 40, expressed as mean (CI). Units μg metal per gram creatinine to control for urine concentration. *p-value <0.05 Paired t-test pre-ethylenediaminetetraacetic acid to post-ethylenediaminetetraacetic acid at baseline. #p-value <0.05. Paired t-test pre-ethylenediaminetetraacetic acid to post-ethylenediaminetetraacetic acid at infusion 40.

Urine Metal	Pre-Ethylenediaminetetraacetic acid baseline	Post-Ethylenediaminetetraacetic acid Baseline	Pre-Ethylenediaminetetraacetic acid infusion 40	Post-Ethylenediaminetetraacetic acid infusion 40
Aluminum (Al)	5.4 (0.7, 10.1)	29.4 (8.4, 50.4)	6.7 (3.5, 10.0)	14.2 (7.4, 21.1)^#^
Antimony (Sb)	0.0 (0.3, 0.5)	0.2 (-0.1, 0.5)	0. (0, 0.1)	0.1 (-0.1, 0.2)
Arsenic (As)	22.6 (13.9, 31.2)	15.6 (11.1, 20.1)^*^	20.3 (2.1, 38.4)	46.3 (-29.5, 122.1)
Barium (Ba)	0.8 (-0.1, 1.7)	1.2 (0.6, 1.8)	1.3 (-1.1, 3.6)	0.4 (0, 0.8)
Bismuth (Bi)	2.0 (-1.9, 5.9)	5.0 (-4.8, 14.8)	2.7 (-3.9, 9.4)	2.3 (-3.3, 7.9)
Cadmium (Cd)	0.6 (0.51, 0.79)	3.6 (2.3, 4.9)^ *^	0.6 (0, 1.3)	4.0 (1.9, 6.0)^#^
Cesium (Cs)	6.5 (4.1, 8.6)	4.6 (2.8, 6.3)^ *^	5.2 (3.4, 7.0)	4.9 (2.7, 7.2)
Gadolinium (Gd)	.5 (-0.3, 1.3)	25.4 (0.7, 50)	0.1 (0, 0.2)	10.1 (-0.3, 20.6)
Lead (Pb)	0.6 (0.3, 0.9)	23 (11.5, 34.5)^ *^	0.2 (0, 0.3)	7.4 (3.0, 11.7)^ #^
Mercury (Hg)	0.5 (0.1, 0.9)	0.4 (0.1, 0.7)	0.5 (0, 1.1)	0.6 (0.0, 1.1)
Nickel (Ni)	3.3 (1.17, 5.4)	8.1 (5.2, 10.9)	3.0 (2.1, 4.0)	6.1 (2.9, 9.2)^ #^
Thallium (Tl)	0.2 (0.1, 0.3)	0.2 (0.1, 0.3)	0.2 (0.1, 0.2)	0.1 (0.1, 0.2)
Tin (Sn)	5.7 (-4.2, 15.6)	74.8 (-70.4, 220)	0.7 (0.5, 0.9)	0.8 (0.6, 1.0)
Tungsten (W)	0.2 (0.1, 0.34)	0.1 (-0.3, 0.6)	0.2 (0.1, 0.3)	0.1 (0.1, 0.2)

Compliance with the study regimen

The median number of infusions completed was 40. Of the 10 subjects, three completed <20 infusions (3, 11, and 19 infusions), and seven participants completed 40 infusions or more (3 completed 40 and 4 completed 50). One subject (004) sought out and received additional, out-of-protocol weekly infusions delivered by a local practitioner on the “off weeks” during the 10 monthly infusion period (study infusion 41-50, after final measurements and quality of life surveys). By the time he had received his 50th study infusion, he had received an additional 25 infusions.

Safety of the intervention

There were 353 intravenous infusions of edetate disodium-based chelation administered to the 10 patients. No treatment-related non-endpoint serious adverse events were reported. In the seven patients completing 40 infusions, the Modification of Diet in Renal Disease (MDRD) estimated glomerular filtration rate did not change at baseline as compared to infusion 40 (mean ± std 77.8 mL/min/1.73 m^2^ ± 17.6 vs. 70.6 mL/min/1.73 m^2^ ± 14.5, p = 0.21) [19].

Cardiovascular endpoints

There were no major adverse cardiovascular events during the infusion phase through the one-year follow-up; nor in those patients receiving 50 infusions in extended follow-up (Table 3).

**Table 3:** Cardiovascular and limb endpoints There were seven patients with wounds (non-healing ulcers of gangrene) at the start of the study. Major cardiovascular endpoints were defined as death from any cause, myocardial infarction, coronary revascularization, or stroke.

	≥ 20 Infusions (n=7)	< 20 Infusions (n=3)	Total Patients (n=10)
Major amputation required	0	2 (66.67%)	2 (66.67%)
Minor amputation required	0	1 (33.3%)	1 (33.3%)
Lower ext. revascularization	1 (14%)	0	1 (14%)
Complete wound healing	5	0	71 %
Major cardiovascular endpoints	0	0	0
Mortality	0	0	0

Limb endpoints

Amputations

Amputations occurred only in the three subjects who were unable to complete the 40 planned edetate disodium-based infusions. These consisted of two major (participants 5 and 7, at infusions 3 and 11) and one minor (participant 8 at infusion 19) amputations. Patient 5 had intractable rest pain unresponsive to medications and underwent urgent amputation after infusion three. Patient 7 had dry gangrene involving three toes proximal to the metatarsal joint. He was hospitalized after 11 infusions, with advancement of his disease, resulting in an above-the-knee amputation of the affected extremity. Patient 8 was unable to continue the protocol due to psychiatric and social issues and had a minor amputation of the affected hallux after 19 infusions. These patients subsequently dropped out of the study, so infusion 20 and 40 data cannot be reported. The demographic and clinical characteristics of patients that had amputations compared with those that did not are shown in Table 4.

**Table 4:** Demographics and baseline urine metals comparing patients with ≥ 20 infusions and < 20 infusions Demographics and baseline urine metals comparing patients with ≥ 20 infusions and < 20 infusions. Categorical values n (%). Continuous values mean (std). Baseline metals mean (CI).

Variables	≥ 20 Infusions (n=7)	< 20 Infusions (n=3)
Demographics		
Age (years) ± std	76 ± 8.3	73 ± 8.1
Baseline Creatinine (mg/dL) ± std	0.92 ± 0.24	1.04 ± 0.29
Sex, female	3 (43%)	1 (33%)
Comorbidities		
Hypertension	7 (100%)	3 (100%)
Diabetes Mellitus	7 (100%)	3 (100%)
Coronary Artery Disease	6 (86%)	1 (33%)
Smoking history	2 (29%)	1 (33%)
Ulcer or gangrene	4 (57%)	2 (66%)
Baseline urine metals μg of metal per g creatinine (95%, CI)		
Pre-infusion Cadmium	0.6 (0.51, 0.79)	1.6 (-1.2, 4.4)
Pre-infusion Lead	0.6 (0.3, 0.9)	0.9 (-0.6, 2.4)
Post-infusion Cadmium	3.6 (2.3, 4.9)	5.8 (1.8, 9.9)
Post-infusion Lead	23 (11.5, 34.5)	8.8 (2.7, 14.9)

No major or minor amputations occurred in the seven patients that completed >20 edetate disodium edetate-based infusions during the 40 infusions period. One patient (01) underwent two revascularizations at infusions 41 and 49.

Wound healing

**Figure 2:**
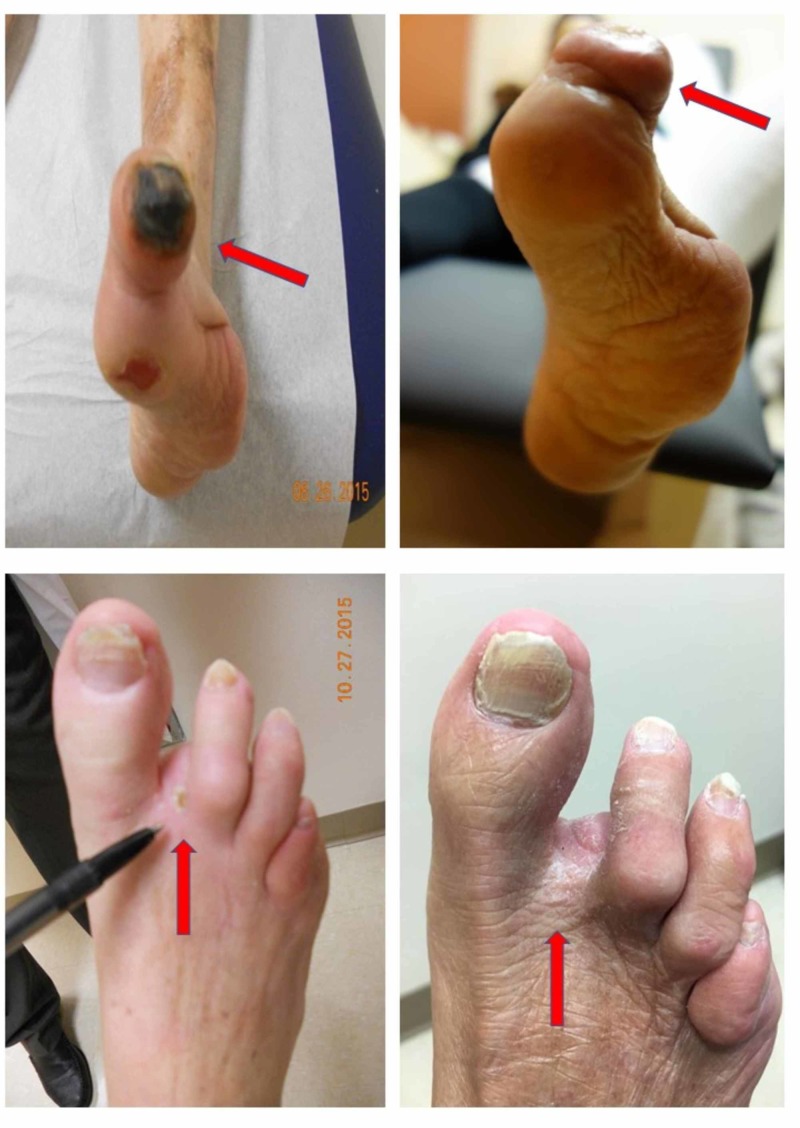
Wound healing Dry gangrene and non-healing ulcer at baseline and infusion 40 for subjects 01 (top row) and 02 (bottom row), demonstrating complete resolution

**Figure 3:**
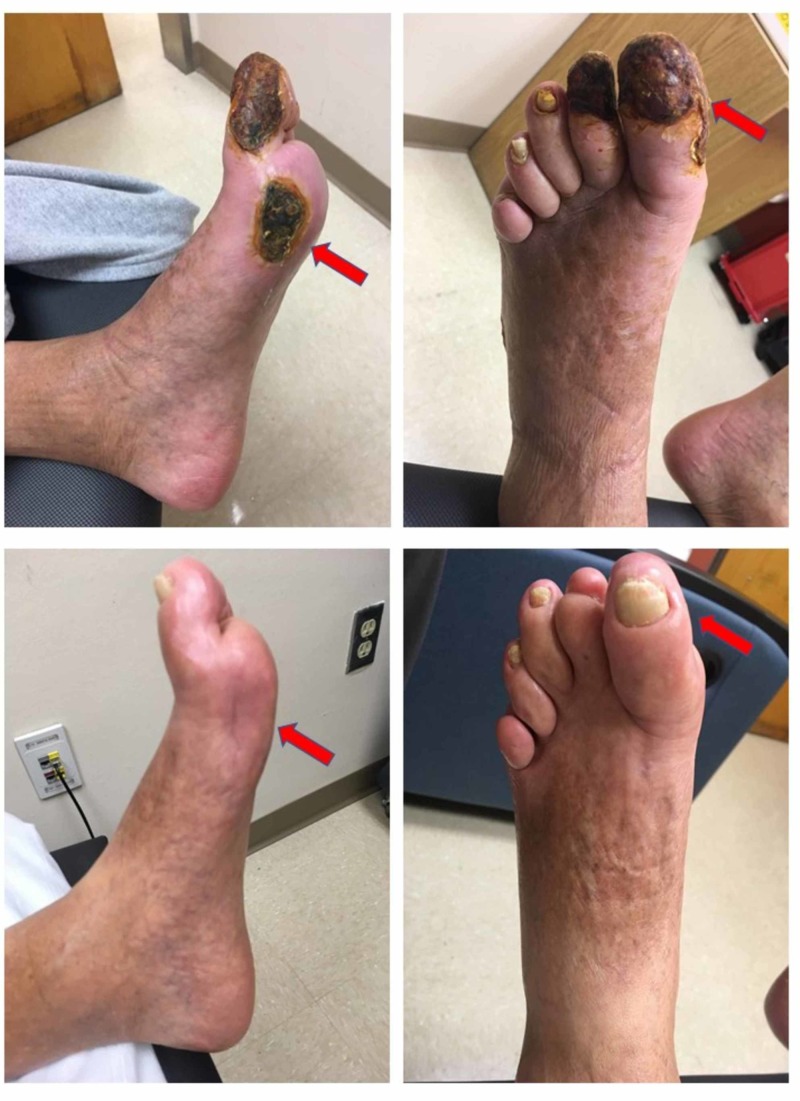
Post-edetate disodium-based chelation Complete resolution of dry gangrene after edetate disodium-based therapy (top baseline, bottom infusion 48)

Five of the seven patients that completed 40 infusions had ulcers or gangrene at enrollment. There were no changes in wound care or secondary prevention before enrollment compared to during the study treatment. In all those patients, ulcers and dry gangrene completely resolved (Figures 2-3) [12]. There were no new active wound infections during the infusion phase.

Quality of life

**Figure 4:**
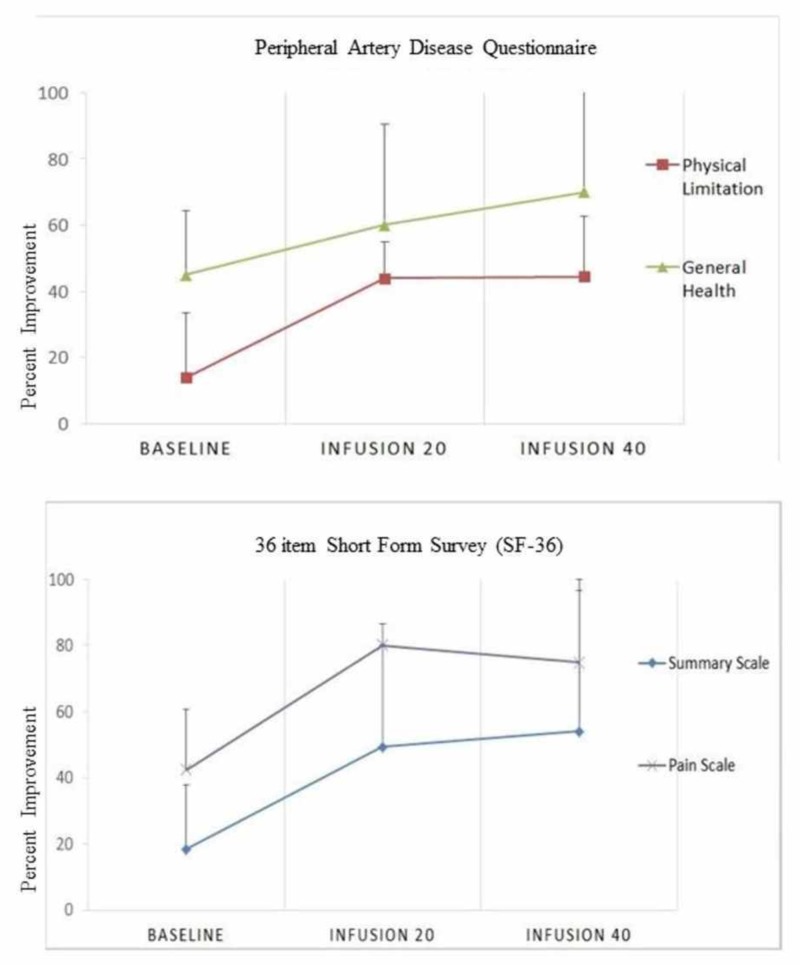
SF-36 and PAD Questionnaire Median and interquartile range of quality of life scores at baseline, 20 and 40 infusions. The graph only includes the seven patients completing 40 infusions. The pain and summary scale pertain to the SF-36 Questionnaire (bottom row). Physical limitation and general health are variables pertaining to the PAD Questionnaire (top row). A lower score on the Y-axis indicates less favorable health or worse function.

Quality of life measured by the SF-36 and PAD disease-specific instruments demonstrated improvements in all categories [15-16]. The analyses include only those seven patients that completed 40 infusions (Figure 4). There was a 76.5% median percent improvement in the SF-36 pain score (median, interquartile range (IQR) 42.5 (10, 50) vs. 75 (46.3, 78.75), p=0.054) from baseline and a 56% median percent improvement (median, IQR 45 (45,64.4) vs 70 (40, 82.5), p=0.089) in general health overall. In the PAD questionnaire, disease-specific quality of life improved by a median percent 351% (13.3 (9.9-30) vs. 60 (33.3-66.4), p=0.018 and summary scale by 193% (18.4 (12.7-30.9) vs. 54 (34-59), p=0.005) (Table 5). 

**Table 5:** Quality of life Quality of life SF-36 and PAD Questionnaires at baseline, infusion 20 and 40 for seven patients completing 40 infusions. Variables expressed as median (interquartile range). ‡Non-parametric, Friedman test.

Questionnaire and scale	Baseline	Infusion 20	Infusion 40	p-value ‡
Peripheral artery disease questionnaire				
Physical limitation	13.9 (5.6-25.0)	44 (36.1-47)	44.4 (33-51.4)	0.236
Symptom stability	33.3 (33.3-41.7)	50 (41.7-58.3)	50 (25-50)	0.058
Symptoms	22.2 (19.45-38.95)	50 (47.2-58.35)	55.6 (17.0-75.0)	0.066
Social limitation	44 (11.1-52.8)	44.4 (41.4-58.4)	53.3 (42-72.25)	0.121
Treatment satisfaction	60 (43.33-76.7)	80 (53.3-80)	60 (53.3-73.3)	0.304
Quality of life	13.3 (9.9-30)	53.3 (50-53.3)	60 (33.3-66.4)	0.018
Summary scale	18.4 (12.7-30.9)	49.4 (48.1-54.6)	54 (34-59)	0.005
36-item short form survey (SF-36)				
Physical function	45 (28.8-60)	58 (37.5-80)	75 (57-80)	0.135
Role functioning/physical	0 (0-35)	75 (17.5-87.5)	100 (55-100)	0.005
Role functioning/emotional	33.3 (0-66.7)	100 (83.4-100)	100 (61.4-100)	0.223
Mental health	45 (32.5-88)	55 (50-88.5)	80 (55-93.5)	0.468
Emotional well-being	80 (74-80)	84 (68-96)	80 (66-96)	0.878
Social functioning	37.5 (12.5-75)	75 (37.5-100)	75 (50-93.8)	0.161
Pain	42.5 (10-50)	80 (53.8-85)	75 (46.3-78.8)	0.054
General health	45 (45-64.4)	60 (44.4-75)	70 (40-82.5)	0.089

Limb perfusion pressures

Skin perfusion pressures (mmHg) in the target vascular bed of the affected foot demonstrate a trend towards improvement after edetate disodium infusions (median (IQR): 22 (17-49), 46 (18-69) and 36 (28-43) at baseline, 20 and 40 infusions, respectively, P=0.06). The median perfusion pressures in the contralateral foot did not significantly change during follow-up (Table 6).

**Table 6:** Skin perfusion pressures (mmHg) at baseline and during follow-up† Changes in skin perfusion pressure (SPP) in the target vascular bed of the affected foot, as well as in the affected and contralateral feet, were measured at baseline, at 20 and 40 infusions. The average skin perfusion pressure for each foot was calculated from the segmental pressures in the medial plantar, lateral plantar, and dorsal foot areas. Values represent median (IQR). †Including only patients with initial and follow-up measurements (n=7). Three individuals did not complete 20 infusions. ‡Friedman analysis of variance (ANOVA).

Vascular beds	Baseline	Number of infusions		P-value‡
		Twenty	Forty	
Contralateral foot	50(42-82)	40(36-66)	51(26-77)	0.4
Target vascular bed	22(17-49)	46(18-69)	36(28-43)	0.06

Outcomes and clinical characteristics of patients that completed 40 vs 20 infusions

**Figure 5:**
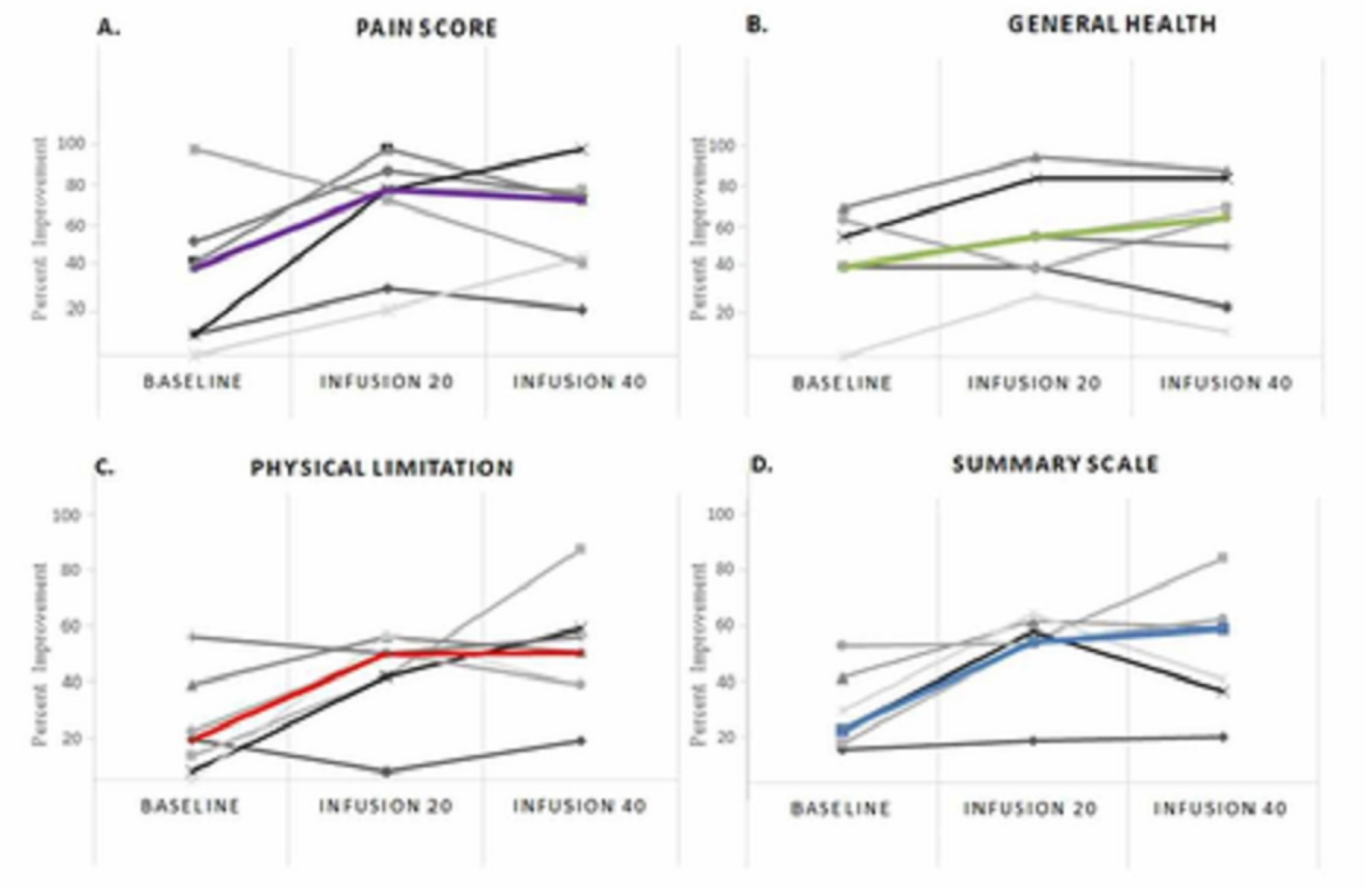
SF-36 and PAD Questionnaires Individual and median (color line) quality of scores for all participants at baseline, 20 and 40 infusions. (A) Pain and (B) General Health pertain to the 36-Item Short Form Survey Questionnaire. (C) Physical limitation and (D) Summary scale are variables pertaining to the Peripheral Artery Disease Questionnaire.

In a non-prespecified, data-derived analysis, we compared patients that completed 40 infusions with the patients undergoing < 20 infusions. Those patients completing < 20 infusions demonstrated numerically greater systolic hypertension (151.3 mm Hg ± 27.4 vs. 142 mm Hg ± 13.7 p=0.12), lower hemoglobin (10 g/dL ± 2.1 vs 12.2 g/dL ± 1.79, p=0.12), and higher level of baseline urinary cadmium (1.6 μg of metal per g creatinine (-1.2, 4.44) vs. 0.4 μg of metal per g creatinine (0.2, 0.5), p=0.18) as compared to those completing 40 infusions. Baseline quality of life measures obtained by the SF-36 and PAD Questionnaires were lower in patients undergoing < 20 infusions as compared to those completing 40 infusions. In particular, pain (Median (IQR); 7.4 (-7.1, 21.9) vs. 37.5 (11.8, 63.2)) and quality of life scores (4.4 (0.1,8.8) vs. 20.9 (8.9, 32.8)) were reduced in those receiving < 20 as compared to those who had ≥ 40 infusions. In the three patients who were not able to complete the 40 planned edetate disodium infusions, two major and one minor amputation occurred as compared to no major or minor amputations in the seven patients completing all infusions. There was no difference in major adverse cardiovascular events between groups (total of 0). See Figure 5 for the baseline values obtained by the SF-36 and PAD Questionnaires.

## Discussion

Clinical studies of edetate disodium chelation to treat PAD have shown mixed results. Case series reported in the distant past are supportive [20]. Small clinical trials reported more recently, but still decades ago, were too small to detect clinical outcome benefits. Softer endpoints, such as ankle-brachial pressure index, walking distance, or pain-free walking were not improved. A Cochrane analysis in 2002 found there was not enough evidence to decide the effectiveness or ineffectiveness of chelation therapy [21]. Our review of this literature is that edetate disodium therapy of PAD is in equipoise, with similar quantities of flawed data falling on either side of the question. On the other hand, a 10-year National Institutes of Health-funded clinical trial, TACT, reported a reduction in cardiovascular events in 1708 post MI patients receiving over 55,000 active or placebo edetate infusions, with large effect size in those with diabetes [10-11]. In a non-prespecified analysis of TACT patients with MI, diabetes, and PAD, we reported a relative reduction of combined cardiovascular events of 48% (p=0.0069) [22].

In view of the indirectly favorable evidence for a treatment effect in PAD from TACT and selected case reports, we sought a signal of benefit for edetate disodium chelation to define whether additional research should be performed. In this 10-patient unblinded pilot study, the seven subjects successfully completing at least 40 edetate disodium-based infusions demonstrated enhanced wound healing and avoided amputation. Quality of life, measured with the SF-36 and the PAD instrument, including pain, physical, and social limitations, markedly improved [15-16]. Furthermore, no major adverse cardiovascular event or treatment-related adverse events were encountered.

Robust epidemiological evidence supports the association of toxic metals with PAD. A population-based cohort study of the National Health and Nutrition Examination Survey (NHANES) data suggest that low-level environmental lead exposure is an important risk factor for cardiovascular disease [23]. Tellez-Plaza et al. demonstrated an association of urine cadmium with PAD [4]. A meta-analysis by Chowdhury et al., encompassing over 300,000 patients, recently reported that lead and cadmium are cardiovascular risk factors with a dose-response relationship [24]. An environment-wide association analysis by Zhuang et al., in 2018, analyzed the NHANES data for predictors of PAD [25]. Cadmium was one out of only four independently predictive variables. These observations, along with favorable outcome data from TACT, including in patients with PAD, suggest that toxic metals may be novel modifiable risk factors for PAD [6,22].

Edetate disodium is an artificial amino acid first synthesized in 1938 and has been in use for the treatment of vascular disease for over 60 years, without, until recently, high-quality evidence in its favor. It is a potent chelator of divalent cations, including vasculotoxic metals, such as lead and cadmium, as well as calcium. Lead and cadmium are widely found in our environment and taken in by humans through food, water, and air [3]. Recent analyses of patients with coronary artery disease have demonstrated that the presence of urine cadmium is highest in patients with CLI, perhaps indirectly supporting the importance of decreasing total body stores of toxic metals [6].

We have proposed that the increased excretion of vasculotoxic metals, such as cadmium and lead, through treatment with edetate disodium-based chelation, may lead to stabilization of diffuse small vessel and microvascular disease, as is typically found in diabetes [26]. In this small cohort, as in a larger cohort [27], a marked increase in urinary excretion of lead and cadmium was observed. Chelation was associated with a ~70% reduction in pre-and post-chelation lead values. There is some evidence suggesting that provoked or post-chelation urine lead is associated with the total body lead burden. This might suggest that the study treatment reduces body stores [28]. In contrast, chelation was not associated with a significant reduction of spontaneous or provoked urinary cadmium during follow-up, suggesting no overall reduction in body stores. Alternatively, edetate disodium chelation may remove cadmium from selective body compartments (e.g. vascular), which we are unable to quantify.

Skin perfusion pressures lower than 40 mmHg signal severe ischemia and reduced chances of spontaneous or post-surgical healing in CLI patients [29]. In the present study, the median skin perfusion pressure in the target vascular bed of the affected foot was severely reduced (22 mmHg), and the median perfusion pressures of both the affected and the contralateral feet were very abnormal (41 and 50 mmHg, respectively). There are no longitudinal studies evaluating changes in skin perfusion pressures over time, and it is assumed that flow measures would decrease over time in parallel with worsening clinical status. In the present study, there was a non-significant trend toward the improvement of flow in the target vascular beds in those individuals who completed all the infusions. Moreover, the overall perfusion pressures in the affected or contralateral feet remained relatively stable during follow-up. Unfortunately, the design and small sample size of this pilot study lacked statistical power to draw definite conclusions on the effect of chelation therapy on vascular flow. Toxic metals may lead to endothelial dysfunction by decreasing nitric oxide production and increasing oxidative stress, leading to vasoconstriction and increased basal tone in the microcirculation [30]. An alternate hypothesis not involving toxic metals is that the infusions simply decalcify advanced atherosclerotic lesions. Edetate disodium is an efficient calcium chelator and was originally FDA-approved for the treatment of hypercalcemia. Ongoing studies, such as TACT2 and TACT3a, will help further explain the interaction of toxic metal chelation, calcified plaque, and vascular outcomes.

This study has many limitations, and one must be wary of inferring unsupported conclusions. This is a small non-randomized pilot study and the results, although promising, could be due to play of chance. The healing rates in ‘no option’ CLI patients treated with placebo in clinical trials range between 10% and 50%. Although unlikely, the responses we observed could have been spontaneous remission of the disease. Skin healing was evident in patients with dry gangrene or non-healing ulcers, but the study does not give us a clear signal as to mechanism. There was a mild signal of improved perfusion in the affected vascular beds but no evidence of improvement or deterioration in overall skin perfusion pressure. This may simply be due to a lack of power to detect small changes in the microcirculation, technical issues with the measurements, or other unknown physiological factors. Lastly, it is possible that non-vascular/flow-related mechanisms, such as metabolic changes provoked by other ingredients in the infusion (i.e. 7 gms ascorbic acid), could also account for some or all of the benefit.

## Conclusions

This pilot study must be interpreted cautiously. It met its goal of providing a signal of benefit and preliminary evidence of safety in this population. TACT3a, a randomized double-blind, placebo-controlled clinical trial, will further test the effects of edetate disodium-based chelation in patients with diabetes and critical limb ischemia.

## References

[1] American Diabetes Association (2018). Centers for Disease Control and Prevention. National Diabetes Statistics Report, 2017. https://www.cdc.gov/features/diabetes-statistic-report/index.html.

[2] (2018). Centers for Disease Control and Prevention. National Diabetes Statistics Report: estimates of diabetes and its burden in the United States, 2017. National Diabetes Statistics Reports.

[3] Nawrot TS, Staessen JA (2006). Low-level environmental exposure to lead unmasked as silent killer. Circulation.

[4] Tellez-Plaza M, Guallar E, Howard BV (2013). Cadmium exposure and incident cardiovascular disease. Epidemiology.

[5] Tellez-Plaza M, Navas-Acien A, Menke A, Crainiceanu CM, Pastor-Barriuso R, Guallar E (2012). Cadmium exposure and all-cause and cardiovascular mortality in the U.S. general population. Environ Health Perspect.

[6] Ujueta F, Arenas IA, Diaz D, Yates T, Beasley R, Navas-Acien A, Lamas GA (2018). Cadmium level and severity of peripheral artery disease in patients with coronary artery disease. Eur J Prev Cardiol.

[7] Lamar CP (1964). Chelation therapy for occlusive arteriosclerosis in diabetic patients. Angiology.

[8] Lamar CP (1966). Chelation endarterectomy for occlusive atherosclerosis. J Am Geriatrics Soc.

[9] Brucknerova O, Tulacek J, Krojzl O (1968). Chelaty v leche uzaverovych tepennych chorob. Vnitrni Lek.

[10] Lamas GA, Goertz C, Boineau R (2013). Effect of disodium EDTA chelation regimen on cardiovascular events in patients with previous myocardial infarction. The TACT randomized trial. JAMA.

[11] Escolar E, Lamas GA, Mark DB (2014). The effect of an EDTA-based chelation regimen on patients with diabetes mellitus and prior myocardial infarction in the Trial to Assess Chelation Therapy (TACT). Circ Cardiovasc Qual Outcomes.

[12] Ujueta F, Arenas IA, Yates T, Beasley R, Diaz D, Lamas GA (2019). Edetate disodium-based treatment in a patient with diabetes and critical limb ischemia after unsuccessful peripheral arterial revascularizations: a case report. Clinical Diabetes.

[13] Lamas GA, Goertz C, Boineau R (2012). Design of the trial to access chelation therapy. Am Heart J.

[14] Larsson J, Strenstrom A, Alpelqvist J, Agardh CD (1995). Decreasing incidence of major amputation in diabetic patients: a consequence of a multidisciplinary foot care team approach. Diabet Med.

[15] Spertus J, Jones P, Poler S, Rocha-Singh K (2004). The Peripheral Artery Questionnaire: a new disease-specific health status measure for patients with peripheral arterial disease. Am Heart J.

[16] Ware JE, Sherbourne D (1992). The MOS 36-item short-form health survey (SF-36). Medical Care.

[17] Friedman M (1937). The use of ranks to avoid the assumption of normality implicit in the analysis of variance. J Am Stat Assoc.

[18] Mayor J, Chung J, Zhang Q, Montero-Baker M, Schanzer A, Conte MS, Mills Sr JL (2019). Using the Society for Vascular Surgery wound, ischemia, and foot infection classification to identify patients most likely to benefit from revascularization. J Vasc Surg.

[19] Levey AS, Bosch JP, Lewis JB, Greene T, Rogers N, Roth D (1999). A more accurate method to estimate glomerular filtration rate from serum creatinine: a new prediction equation. Modification of Diet in Renal Disease Study Group. Ann Intern Med.

[20] Hancke C, Flytlie K (1993). Benefits of EDTA chelation therapy in arteriosclerosis: a retrospective study of 470 patients. Int J Adv Med.

[21] Dans AL, Tan FN, Villarruz-Sulit EC (2002). Chelation therapy for atherosclerotic cardiovascular disease. Cochrane Database Syst Rev.

[22] Ujueta F, Arenas IA, Escolar E (2019). The effect of EDTA-based chelation on patients with diabetes and peripheral artery disease in the Trial to Assess Chelation Therapy (TACT). J Diabetes Complications.

[23] Lanphear BP, Rauch S, Auinger P, Allen RW, Hornung RW (2018). Low-level lead exposure and mortality in US adults: a population-based cohort study. Lancet Public Health.

[24] Chowdhury R, Ramond A, O’Keeffe LM (2018). Environmental toxic metal contaminants and risk of cardiovascular outcomes: systematic review and meta-analysis. BMJ.

[25] Zhuang X, Ni A, Liao L (2018). Environmental-wide association study to identify novel factors associated with peripheral arterial disease: evidence from the National Health and Nutrition Examination Survey (1999-2004). Atherosclerosis.

[26] Lamas GA, Navas-Acien A, Mark DB, Lee KL (2016). Heavy metals, cardiovascular disease, and the unexpected benefits of chelation therapy. J Am Coll Cardiol.

[27] Arenas IA, Navas-Acien A, Ergui I, Lamas GA (2017). Enhanced vasculotoxic metal excretion in post-myocardial infarction patients following a single edetate disodium-based infusion. Environ Res.

[28] Bradberry S, Sheehan T, Vale A (2009). Use of oral dimercaptosuccinic acid (succimer) in adult patients with inorganic lead poisoning. QJM-Int J Med.

[29] Pan X, You C, Chen G, Shao H, Han C, Zhi L (2018). Skin perfusion pressure for the prediction of wound healing in critical limb ischemia: a meta-analysis. Arch Med Sci.

[30] Qiuan Z, Xiaofei L, Qingjiao N, Mao B, Pan X (2017). Metabolic profiling in association with vascular endothelial cell dysfunction following non-toxic cadmium exposure. Int J Mol.

